# ProkSeq for complete analysis of RNA-Seq data from prokaryotes

**DOI:** 10.1093/bioinformatics/btaa1063

**Published:** 2020-12-26

**Authors:** A K M Firoj Mahmud, Nicolas Delhomme, Soumyadeep Nandi, Maria Fällman

**Affiliations:** Laboratory for Molecular Infection Medicine Sweden (MIMS), Umeå Centre for Microbial Research (UCMR), Department of Molecular Biology, Umeå University, SE-901 87 Umeå, Sweden; Department of Forest Genetics and Plant Physiology, Umeå Plant Science Centre (UPSC), Swedish University of Agricultural Sciences, Umeå, Sweden; Amity Institute of Integrative Sciences and Health, Amity University Haryana, Gurgaon, 122413 Haryana, India; Laboratory for Molecular Infection Medicine Sweden (MIMS), Umeå Centre for Microbial Research (UCMR), Department of Molecular Biology, Umeå University, SE-901 87 Umeå, Sweden

## Abstract

**Summary:**

Since its introduction, RNA-Seq technology has been used extensively in studies of pathogenic bacteria to identify and quantify differences in gene expression across multiple samples from bacteria exposed to different conditions. With some exceptions, tools for studying gene expression, determination of differential gene expression, downstream pathway analysis and normalization of data collected in extreme biological conditions is still lacking. Here, we describe ProkSeq, a user-friendly, fully automated RNA-Seq data analysis pipeline designed for prokaryotes. ProkSeq provides a wide variety of options for analysing differential expression, normalizing expression data and visualizing data and results.

**Availability and implementation:**

ProkSeq is implemented in Python and is published under the MIT source license. The pipeline is available as a Docker container https://hub.docker.com/repository/docker/snandids/prokseq-v2.0, or can be used through Anaconda: https://anaconda.org/snandiDS/prokseq. The code is available on Github: https://github.com/snandiDS/prokseq and a detailed user documentation, including a manual and tutorial can be found at https://prokseqV20.readthedocs.io.

**Supplementary information:**

Supplementary data are available at *Bioinformatics* online.

## 1 Motivation

The advancement of massive parallel sequencing and dramatic reduction in sequencing costs have made deep sequencing of RNA (RNA-Seq) a primary tool for identifying and quantifying RNA transcripts. Today RNA-Seq is widely used to analyse bacterial gene expression in studies that aim to identify drug targets, predict novel gene regulatory mechanisms, etc. Such studies often require profound knowledge of both computational data handling and biology. There are some stand-alone pipelines and tools that require only moderate knowledge of bioinformatics (Delhomme *et al.*, 2012; Prieto and Barrios, 2020), but these are not designed for analyses of bacterial gene expression.

Prokaryotic RNA-Seq analysis is challenging because most available RNA-Seq packages assume the input data reflect eukaryotic gene structures, which in many aspects differ from those of prokaryotes (Johnson *et al.*, 2016). Bacterial transcripts do not have introns and are not alternatively spliced; therefore, using an aligner developed to consider splice junctions often increases falsely assigned reads in the genome (Magoc *et al.*, 2013). Moreover, unlike in eukaryotes, under specific stresses, the expression of almost all prokaryotic genes can change (Creecy and Conway, 2015). Furthermore, quality trimming, adapter removal and normalization of skewed data are often required for prokaryotic data due to variations in experimental setups, the presence and overexpression of plasmid genes and differences in RNA-Seq protocols (Magoc *et al.*, 2013; McClure *et al.*, 2013).

**Fig. 1.**
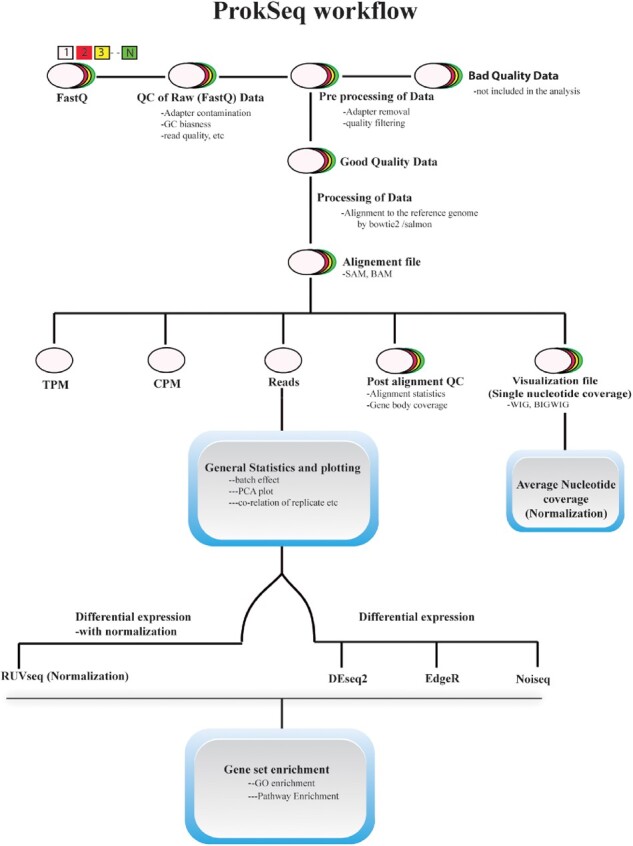
Workflow of ProkSeq showing major steps and tools

Although there are a few software packages available for prokaryotes that can facilitate the analysis of RNA-Seq data, such as SPARTA (Johnson *et al.*, 2016), EDGE-pro (Magoc *et al.*, 2013) and RockHopper (McClure *et al.*, 2013), all require substantial knowledge of data handling. Therefore, to reduce human intervention in conducting RNA-Seq data analysis for prokaryotes, we developed ProkSeq, a fully automated command-line based workflow by integrating various available tools and built-in functions written in Python. ProkSeq integrates short read aligner bowtie2 (Langmead and Salzberg, 2012) with its default parameter as well as Salmon (Berghoff et al., 2017) as an option for (pseudo-)alignment. It provides normalized expression value to compare within and between samples, options to remove unwanted variation (RUV) (Risso *et al.*, 2014) and average nucleotide count normalization for differential expression (Creecy and Conway, 2015). In addition, ProkSeq supports downstream Gene Ontology (GO) (Gene Ontology Consortium, 2008) and KEGG pathway enrichment analyses (Kanehisa and Goto, 2000). ProkSeq processes RNA-Seq data from quality control steps to pathway enrichment analysis of differentially expressed genes (Fig. 1). It provides a wide variety of options for differential expression, normalized expression and visualization, and produces figures. Reduced human intervention and multithreading feature makes the use of ProkSeq less time consuming than the sequential application of separate tools, which often requires reformatting data.

## 2 Implementation

ProkSeq runs in a Linux-based command-line environment and depends on user-defined parameters and sample files. The tools used in the pipeline are set with their default parameters. However, more advanced users can adjust the parameters of the tools to control the functionality. The sample file indicates the names of the fastq files to be included in the analysis, and also defines the experimental classes, such as treatment and control samples. ProkSeq first checks the quality of reads and filters out low-quality reads using FastQC (http://www.bioinformatics.babraham.ac.uk/projects/fastqc) and afterQC (Chen *et al.*, 2017). It maps the reads to the reference genome using bowtie2 and its default parameters for both single and paired-end reads. ProkSeq then generates a report on the alignment quality for each library, as both figures and text, providing information about coverage uniformity, distribution along protein-coding sequences, and 5' and 3' UTR regions, as well as the read duplication rate and strand specificity generated by RSeQC (Wang *et al.*, 2012). Total reads per gene are calculated with featureCounts (Liao *et al.*, 2014), which provides a high efficiency of read assignments across the genome. ProkSeq also calculates normalized gene expression values for each gene, in the form of transcripts per million (TPM) and counts per million (CPM) (Wagner *et al.*, 2012). The formulas by which these are calculated are explained in the Supplementary Methods (S1).

ProkSeq integrates several tools for differential expression analysis, such as DESeq2 (Love *et al.*, 2014), edgeR (Robinson *et al.*, 2010) and NOISeq (Tarazona *et al.*, 2015). For downstream analysis of differentially expressed genes, ProkSeq uses GO enrichment and pathway enrichment by integrating clusterProfiler (Yu *et al.*, 2012). Reports on pre- and post-alignment quality statistics and graphical visualization are created in pdf and HTML formats. One important unique feature of ProkSeq is the integration of RUV normalization and average nucleotide count methods for skewed data (Creecy and Conway, 2015; Zhu *et al.*, 2019). Furthermore, the package generates a single-nucleotide resolution wiggle file for visualization in any genome browser. ProkSeq generates graphics and figures at every step of data analysis to give the user more confidence in and understanding of their data. The methods are described in detail in the Supplementary Methods (S1).

## 3 Discussion

ProkSeq has been designed to meet researchers with moderate bioinformatics knowledge for analysing RNA-Seq data in a reliable and time-efficient way. RNA-Seq data can provide much more information than simply the differential expression of known coding sequences. Exploring RNA-Seq reads to single-nucleotide resolution across the genome can provide information about biological events other than gene expression. ProkSeq offers easy access to genome-wide visualization of RNA-Seq data. Visualization of read mapping will reveal expression from unannotated genomic regions and intergenic regions, including 5’ and 3’ UTRs, which is of great interest in relation to novel transcriptional and translational regulation. Other tools for revealing this type of information that are available today (Supplementary Table S1) usually require substantial competence in bioinformatics and lack some of the options available in ProkSeq. Furthermore, integration of Salmon in the process gives the user one of the most up-to-date methods of estimating transcript abundance. Salmon uses a realistic model of RNA-Seq data that takes into account not only experimental attributes but also biases commonly observed in RNA-Seq data (Bergoff et al., 2017). Users can quickly extract transcript abundance and subsequent differential expression data by opting to use salmon.

ProkSeq provides an option for batch effect identification and normalization. An essential difference between eukaryotes and prokaryotes that can cause problems when analysing prokaryotic gene expression using tools optimized for analyses of eukaryotic cells is the relative number of differentially expressed genes. Most often, tools such as DESeq2, edgeR and Limma (Dillies *et al.*, 2013) are designed with the assumption that the number of genes is constant in eukaryotes. But in prokaryotes, the expression of the majority of genes can be altered under specific stress conditions (Berghoff *et al.*, 2017; Creecy and Conway, 2015). To address this bias, ProkSeq normalizes the data at the level of nucleotide base count making the data comparable across samples. ProkSeq provides two normalization options that can handle differential expression analyses of this type of data, which are described in detail in the Supplementary Methods (S1).

The built-in automatic sequential handling of the data from differential gene expression analysis to downstream functional analyses allows researchers to focus on complex biological mechanisms instead of tackling bioinformatics obstacles. The flexibility that comes with built-in options for certain steps and the visualization of mapped reads across genomes opens a path to new discoveries in gene regulation as well as in RNA biology.

## Supplementary Material

btaa1063_Supplementary_Data
