# Supplementary material for: ProkSeq for complete analysis of RNA-Seq data from prokaryotes
Source: Bioinformatics. 2020 Dec 26;37(1):126–8. doi: 10.1093/bioinformatics/btaa1063 (PMC8034529; doi:10.1093/bioinformatics/btaa1063)
Supplement: btaa1063_Supplementary_Data [file btaa1063_supplementary_data.docx]

**Supplementary Methods S1**

**Quality control of reads and filtering:**

Quality control of pre-aligned data is very important for reducing noise in RNA-Seq data and getting biologically insightful results. Here we integrated the widely used fastQ quality checking tools FastQC (http://www.bioinformatics.babraham.ac.uk/projects/fastqc) and afterQC (Chen, et al., 2017), which can filter out poor-quality data. Because afterQC implements trimming globally by allowing all the reads to be trimmed identically, we have included it as a default setting in ProkSeq so that inexperienced users do not have to think about trimming criteria and bias. In this step, poor-quality reads are eventually filtered out and contaminating adapter sequences are removed. After alignment to the reference genome it is important to get an overview of some of the information in order to interpret the data correctly. RseQC (Wang, et al., 2012) and some built-in functions have been integrated to generate a quality report for each library after alignment

**Mapping to the reference genome:**

ProkSeq maps the reads to the reference genome using bowtie2 (Langmead and Salzberg, 2012) with its default parameters for both single and paired-end reads. Users have the flexibility to change the parameters by changing the parameter.txt file. Bowtie2 was chosen for read mapping due to its flexibility, sensitivity, and lower rate of false mapping (Magoc, et al., 2013) compared with other available short-read aligners used to map bacterial genomes, such as segemehl (Hoffmann, et al., 2009), BBmap (Roy and Chanfreau, 2020), etc.

**Calculated read count and expression per gene:**

ProkSeq calculates total reads per gene with featureCounts (Liao, et al., 2014), which was chosen for its short running times and high efficiency in assigning reads to different genomic features. Most differential expression analysis methods are based on the total number of reads in genes but in some cases when user wants to if the genes are highly expressed or not among the experiment, it is useful to apply normalized gene expression values. For this reason ProkSeq has been designed to calculate counts per million (CPM) and transcripts per million (TPM)(Wagner, et al., 2012). The formulas by which CPM and TPM are calculated are as follows:

CPM = (number of reads mapped to a gene / total number of mapped reads of a sample) × 10^6^

TPM= (number of reads mapped to a gene/ gene length) / ∑ (reads mapped to a gene/gene length) × 10^6^

**Differential expression analysis:**

It has been demonstrated in several studies that assumptions of equal means and variances do not adequately accommodate RNA-Seq data, which are over-dispersed and exhibit great variability between biological replicates (Anders and Huber, 2010). To improve the predictability of differential gene expression, several tools and methodologies have been developed. The most commonly used tools for differential expression analyses are DESeq2 and edgeR, which can all predict differential expression reliably with a limited number of biological replicates. These DE tools mentioned as well as NOISeq are integrated into ProkSeq, the latter being useful for samples with fewer biological replicates (Tarazona, et al., 2015). In the most recent DESeq2 version (>1.16), the shrinkage of log2 fold change (LFC) estimates is not performed by default. ProkSeq perform it by default and saves the result in a file so users can compare with or without LFC shrinkage. Lowly expressed genes tend to have relatively higher levels of variability and LFC shrinkage can reduces it by using information from all genes to generate more accurate estimates (Zhu, et al., 2019)

**Normalization and differential expression for skewed data:**

Commonly used differential expression methods assume that the numbers of up- and down-regulated genes are balanced between different conditions (Berghoff, et al., 2017). Therefore, these methods might perform poorly when the expression of up to one-fourth of genes is differentially expressed in bacteria in certain biological conditions (Khil and Camerini-Otero, 2002; Kroger, et al., 2013; Yang, et al., 2018). As a consequence, there is a risk that some genes could be wrongly predicted as differentially expressed. To avoid this, two other normalization methods are included in ProkSeq. These are well-established methods used for analysing bacterial gene expression: (1) Normalization by using factor analysis of control genes by RUVSeq package (Risso, et al., 2014). ProkSeq provide the option for the users to use their own control gene list or prediction of in-silico empirical negative control genes from the RNA-Seq data. ProkSeq by default predict 100 least differential expressed genes and used as a negative control to remove unwanted variation from the data and successively calculates differential expression by RUVg function in RUVSeq DESeq2. Risso, et al., 2014 showed that RUVSeq effectively reduces library preparation effects without weakening the sample versus control effect by using *in silico* empirical control genes. Similar approach has been applied to normalize for bacterial RNA-Seq data of extreme conditions and result shows the importance of use control genes for normalization (Berghoff, et al., 2017).

(2) Gene-specific read alignments, in which each value is expressed as a base count per billion bases counted (Creecy and Conway, 2015). Normalize base count method is a variant of the total count approach that use for normalizing gene-specific read alignments. In case of extreme conditions where data are presumed to have larger variation between two groups, statistical significance of differential expression of a gene can be further evaluated by using average normalized base count data as shown by Creecy et al., 2015. Average base counts of individual transcriptional features make all samples directly comparable when two data sets have higher variation because of the experimental setup like logarithmic phase versus stationary phase (Creecy and Conway, 2015).

**Gene Ontology (GO) and KEGG pathway enrichment analysis:**

ProkSeq uses GO enrichment, and KEGG pathway enrichment by integrating clusterProfiler (Yu, et al., 2012). ProkSeq automatically selects the differential expressed genes from the analysis with a cut off p adjusted value >.05 and log2fold change >2(+\-) as a default for the GO enrichment and KEGG pathway analysis. User has the flexibility to change this cut off in the parameter file. ProkSeq uses the option for GO enrichment of non-model organisms in clusterProfiler (Yu, et al., 2012). GO annotation file for the organism can be downloaded and converted to require format by “GOannotation.sh” which is an added feature in ProkSeq. For KEGG pathway enrichment analysis, ProkSeq uses “enrichKEGG” command that allows to search enriched pathways in KEGG database.

**Data visualization and plots:**

To obtain a view of transcriptome dynamics as a whole, and especially hints about regulatory events such as ribo-switches, small RNAs, and transcriptional start sites, data visualization can be helpful. In addition, visualization also allows scanning of whole genomes to reveal read distributions, which can indicate the expression of intergenic regions and 5´ or 3´ untranslated regions, and alternative transcriptional start sites. ProkSeq provides a default option for data visualization in the form of bigwig files, library depth normalized wig files as well as figures in pdf formats at different steps in the data handling process (Fig. 1). User can visualize bigwig and normalized wig files by using any genomic browser such as integrative genomic viewer (IGV).

**References**

Anders, S. and Huber, W. Differential expression analysis for sequence count data. *Genome Biol* 2010;11(10):R106.

Berghoff, B.A.*, et al.* RNA-sequence data normalization through in silico prediction of reference genes: the bacterial response to DNA damage as case study. *BioData Min* 2017;10:30.

Chen, S.*, et al.* AfterQC: automatic filtering, trimming, error removing and quality control for fastq data. *BMC Bioinformatics* 2017;18(Suppl 3):80.

Creecy, J.P. and Conway, T. Quantitative bacterial transcriptomics with RNA-seq. *Curr Opin Microbiol* 2015;23:133-140.

Hoffmann, S.*, et al.* Fast mapping of short sequences with mismatches, insertions and deletions using index structures. *PLoS Comput. Biol.* 2009;5(9):e1000502.

Khil, P.P. and Camerini-Otero, R.D. Over 1000 genes are involved in the DNA damage response of Escherichia coli. *Molecular Microbiology* 2002;44(1):89-105.

Kroger, C.*, et al.* An infection-relevant transcriptomic compendium for Salmonella enterica Serovar Typhimurium. *Cell Host Microbe* 2013;14(6):683-695.

Langmead, B. and Salzberg, S.L. Fast gapped-read alignment with Bowtie 2. *Nat Methods* 2012;9(4):357-359.

Liao, Y., Smyth, G.K. and Shi, W. featureCounts: an efficient general purpose program for assigning sequence reads to genomic features. *Bioinformatics* 2014;30(7):923-930.

Magoc, T., Wood, D. and Salzberg, S.L. EDGE-pro: Estimated Degree of Gene Expression in Prokaryotic Genomes. *Evol Bioinform Online* 2013;9:127-136.

Risso, D.*, et al.* Normalization of RNA-seq data using factor analysis of control genes or samples. *Nat Biotechnol* 2014;32(9):896-902.

Roy, K.R. and Chanfreau, G.F. Robust mapping of polyadenylated and non-polyadenylated RNA 3' ends at nucleotide resolution by 3'-end sequencing. *Methods* 2020;176:4-13.

Tarazona, S.*, et al.* Data quality aware analysis of differential expression in RNA-seq with NOISeq R/Bioc package. *Nucleic Acids Res* 2015;43(21):e140.

Wagner, G.P., Kin, K. and Lynch, V.J. Measurement of mRNA abundance using RNA-seq data: RPKM measure is inconsistent among samples. *Theory Biosci* 2012;131(4):281-285.

Wang, L., Wang, S. and Li, W. RSeQC: quality control of RNA-seq experiments. *Bioinformatics* 2012;28(16):2184-2185.

Yang, B.*, et al.* Global transcriptional regulation by BirA in enterohemorrhagic Escherichia coli O157:H7. *Future Microbiol* 2018;13:757-769.

Zhu, A., Ibrahim, J.G. and Love, M.I. Heavy-tailed prior distributions for sequence count data: removing the noise and preserving large differences. *Bioinformatics* 2019;35(12):2084-2092.

**Supplementary Table S1: Comparative features of Tools commonly used for Bacterial RNA-Seq data analysis**

| **Tools/pipeline** | **PreQC** | **Quality**  **trimming** | **Alignment** | **PostQC** | **Normalized Gene**  **Expression** | **Batch Effect** | **Differential**  **expression** | **Downstream**  **Analysis** | **Genome coverage file** | **Normalized genome coverage file** | **Plot graph** | **Other features** |
| --- | --- | --- | --- | --- | --- | --- | --- | --- | --- | --- | --- | --- |
| **RockHopper** | NO | NO | RockHopper | NO | NO | NO | RockHopper  (DESeq algorithm) | NO | YES | NO | NO | De novo, assembly, operon predictions |
| **SPARTA** | YES | YES | bowtie | NO | NO | YES | edgeR | NO | NO | NO | YES |  |
| **EDGE-pro** | NO | NO | bowtie2 | NO | RPKM | NO | NO | NO | YES | NO | NO | Consider overlapping genes during read counts |
| **READemption** | YES | YES | segmehel | YES | TPM, RPKM and TNOAR | NO | DESeq2 | NO | YES | YES | YES |  |
| **ProkSeq** | YES | YES | bowtie2 | YES | TPM,CPM | YES | DESeq2,edgeR,  NoiSeq | YES | YES | YES | YES |  |

**NO= Not available**

**YES = Available**
